# Drug-Induced Acute Kidney Injury: A Study from the French Medical Administrative and the French National Pharmacovigilance Databases Using Capture-Recapture Method

**DOI:** 10.3390/jcm10020168

**Published:** 2021-01-06

**Authors:** Anne-Lise Rolland, Anne-Sophie Garnier, Katy Meunier, Guillaume Drablier, Marie Briet

**Affiliations:** 1 Département d’Information Médicale, Centre Hospitalo-Universitaire d’Angers, 49100 Angers, France; anne-lise.rolland@etud.univ-angers.fr (A.-L.R.); katy.meunier@chu-angers.fr (K.M.); 2 Service de Néphrologie-Dialysis-Transplantation, Centre Hospitalo-Universitaire d’Angers, 49100 Angers, France; annesophie.garnier@chu-angers.fr; 3 Laboratoire MitoVasc, INSERM U1083, CNRS UMR 6015, Université d’Angers, 49100 Angers, France; 4 Service de Pharmacologie-Toxicologie et Centre Régional de Pharmacovigilance, Centre Hospitalo-Universitaire d’Angers, 49100 Angers, France; guillaume.drablier@chu-angers.fr

**Keywords:** drug-induced acute kidney injury, capture-recapture method, KDIGO, French pharmacovigilance database, French medical information system program, notification rate

## Abstract

Background: Acute kidney injury (AKI) is a public health concern. Among the pathological situations leading to AKI, drugs are preventable factors but are still under-notified. We aimed to provide an overview of drug-induced AKI (DIAKI) using pharmacovigilance and medical administrative databases Methods: A query of the PMSI database (French Medical Information System Program) of adult inpatient hospital stays between 1 January 2017 and 31 December 2018 was performed using ICD-10 (International Classification of Diseases 10th revision) codes to identify AKI cases which were reviewed by a nephrologist and a pharmacovigilance expert to identify DIAKI cases. In parallel, DIAKIs notified in the French Pharmacovigilance Database (FPVDB) were collected. A capture-recapture method was performed to estimate the total number of DIAKIs. Results: The estimated total number of DIAKIs was 521 (95%CI 480; 563), representing 20.0% of all AKIs. The notification was at a rate of 12.9% (95%CI 10.0; 15.8). According to the KDIGO classification, 50.2% of the DIAKI cases were stage 1 and 49.8% stage 2 and 3. The mortality rate was 11.1% and 9.6% required hemodialysis. Conclusion: This study showed that drugs are involved in a significant proportion of patients developing AKI during a hospital stay and emphasizes the severity of DIAKI cases.

## 1. Introduction

Acute kidney injury (AKI) is an increasing health burden worldwide in terms of morbidity, mortality, and economic impact [1]. The hospital incidence rate for AKI in adults is 22% and the pooled AKI-associated mortality rates are 24% according to the international meta-analysis performed by Susantitaphong et al. [2]. In addition, AKI is an increasingly recognized risk factor for chronic kidney disease (CKD) and end-stage renal disease [3,4].

Many types of pathological situations may cause AKI including sepsis, critical illness, circulatory shock, radiocontrast agents, and nephrotoxic drugs. Among them, exposure to nephrotoxic drugs or radiocontrast agents are preventable factors [5,6]. A deeper understanding will allow us to prevent the risk of drug-induced acute kidney injury (DIAKI).

The pharmacovigilance system is based on spontaneous notifications by clinicians. According to a systematic review of under-reporting of adverse drug reaction (ADRs), the median rate of reporting is around 6% [7]. In most cases, the ADRs reported are those that are serious and/or unknown. The current pharmacovigilance system cannot therefore provide an exhaustive overview of ADRs occurring in hospitals. In addition to the pharmacovigilance data, epidemiological studies have shown that the French Medical Information System Program (PMSI) is an efficient tool to detect ADRs [8,9]. Using these two databases, the use of the capture-recapture method could provide an estimation of the prevalence of all ADRs of interest such as DIAKIs [10].

The aims of the present study, based on the PMSI and the French Pharmacovigilance Database (FPVDB), is to provide an overview of DIAKI in terms of prevalence, notification, clinical presentation, and involved drugs, and to define the best ICD-10 code association for DIAKI detection.

## 2. Materials and Methods

### 2.1. Data Sources

This retrospective study was conducted in the University Hospital of Angers (Pays de la Loire, France) with a total number of hospital admissions around 100,000 per year. Two data sources were used: the PMSI database of the Angers University Hospital and the FPVDB. The ethical committee of the Angers University Hospital and the French data protection authority (CNIL) approved the study (decision novar19-0067v0).

The French PMSI generates diagnosis-related groups containing administrative data (name, gender, birthdates, dates of hospital admission and discharge), all the diagnoses coded following the International Classification of Diseases 10th revision (ICD-10) and the procedures coded from the Common Classification of Medical Procedures (CCMP), reported by the physicians for each hospital stay.

The FPVDB includes all adverse drug reaction (ADR) reports notified to a French regional pharmacovigilance center since 1985 by healthcare professionals and patients. ADR notification by healthcare professionals to the pharmacovigilance system is mandatory by law in France especially for serious and/or unexpected ADR. The registered reports are anonymous. However, the original medical records are archived in the pharmacovigilance center of the university hospital that allows the coupling of the two databases.

### 2.2. Case Definition

We investigated all adult cases of acute kidney injury (AKI) that were managed during a hospital stay with an entry date between 1 January 2017 and 31 December 2018. The definition of the KDIGO (Kidney Disease Improving Global Outcomes) 2012 guidelines were used as diagnostic criteria. To summarize, AKI diagnosis was retained in the presence of one of the following three criteria: i/an increase in serum creatinine greater than 1.5 times the proven or presumed base creatinine in the previous seven days, ii/an increase in serum creatinine greater than or equal to 26.5 μmol/Lin 48 h, or iii/a diuresis of less than 0.5 mL/kg/hour over a minimum period of six h [11].

### 2.3. Collection of Drug-Induced Acute Kidney Injury (DIAKIs) Cases

The request made from the PMSI of the Angers University Hospital was based on the following criteria: All stays with an entry date between 1 January 2017 and 31 December 2018, adults aged 18 and over, with an ICD-10 code [12] as the main diagnosis or as associated diagnosis recorded: N17.0, N17.1, N17.2, N17.8, N17.9, N99.0, or R39.2 (Table S1). The following variables were automatically extracted: sex, length of stay (days), date of entry, date of discharge, age of the patient at hospital discharge, mode of patient discharge (death, transfer, return home), passage to a medical or surgical resuscitation unit. Recorded codes of renal replacement therapy (RRT) according to the French Common Classification of Medical Procedures (CCMP) were JVJF002 (RRT by intermittent hemodialysis, hemofiltration, or hemodiafiltration for AKI), JVJF005 (RRT by continuous hemodialysis, hemofiltration, or hemodiafiltration for AKI) and JVJB002 (RRT by peritoneal dialysis for AKI) [13]. The following stays were excluded: stays with an act CCMP of renal transplantation, stays where the patient already had end-stage renal disease supplemented by dialysis, stays with voluntary drug intoxication, and stays with drug overdose. When several stays referred to the same AKI episode, the first stay or the stay with a notification to the RPVC was selected. All cases were reviewed by a nephrologist and a pharmacovigilance expert to check if the AKI episodes met the definition of the KDIGO. All stays with an episode of AKI not meeting this definition were excluded. All included stays were reviewed by a pharmacovigilance expert and/or a nephrologist to investigate whether a drug-related cause was suspected in the onset of the AKI episode according to observations, reports, and letters related to the stay. For each included stay, the PMSI coding was examined: the AKI ICD-10 codes used (N17, N99, or R39.2) and the presence of the ICD-10 code for adverse drug reaction (ADR) (T88.1, codes Y40, and codes Y50).

We extracted all DIAKIs from the FPVDB using the following criteria: notification by a health professional of the Angers University Hospital, ADR occurring between 1 January 2017, and 31 December 2018, adult 18 years of age or older, including the LLT (Lowest Level Term) of the Medical Dictionary for Regulatory Activities (MedDRA): “Creatinine increased”, “Serum creatinine increased”, “Blood creatinine increased”, “Renal function aggravated”, “Renal failure”, “Renal insufficiency”, “Acute renal failure”, “Acute renal insufficiency”, “Renal failure aggravated”, “Renal insufficiency aggravated”, “Acute on chronic renal failure”, “Renal failure acute on chronic”, or “Anuric renal failure” [14]. Next, for each selected notification, we verified that the definition of the AKI met the definition provided by the KDIGO guidelines and if there was a hospital stay corresponding to the case recorded in the PMSI by matching. Cases, where AKI was secondary to drug-induced diarrhea, were excluded.

### 2.4. Estimation of the Prevalence of DIAKI Cases in Hospital Stays Using Capture-Recapture Method

The capture-recapture method was used to estimate the total number of DIAKIs throughout the study, in adults treated at the Angers University Hospital. This method estimates the total number of individuals in a population from several incomplete sources (two or more) using the number of individuals counted in each source and the common number from one source to another [15]. In the present study, two incomplete data sources were used: the PMSI database and the FPVDB. These sources provide information about the DIAKI cases identified in the FPVDB (capture), the number of DIAKI cases identified in the PMSI database (recapture), and the number of cases common with two sources.

The conditions for using the capture-recapture method were checked: i/all cases are real cases and the case definition must therefore be rigorously the same from each source, ii/homogeneity of capture between all sources must be equal, iii/the population studied must be a closed population, iv/the period and the geographical area are the same, v/all common cases are identified and are true duplicates, and vi/the sources must be independent. We applied the maximum likelihood estimator and the adjustment following the Chapman method [16] to the two sources of data (FPVBD and PMSI) to obtain the estimated total number of DIAKI. This method was shown to have the optimal properties under a wide range of conditions and to decrease the small-sample bias to the maximum likelihood estimator [8,17].

From the estimate of the total number of DIAKIs, the number of DIAKIs that were identified by neither of the two sources was deducted. For both sources, we determined the performance of the query—the percentage of DIAKIs detected, divided by total cases extracted—and exhaustiveness—the number of DIAKIs detected, divided by the estimated total number of DIAKIs. Finally, from the number of DIAKIs reported to the FPVDB and the estimated total number of DIAKIs, we calculated the notification rate of DIAKIs.

### 2.5. Description and Analysis of DIAKI Cases

For each DIAKI case, we collected data about sex, age, length of stay (days), death, RRT, intensive care unit hospitalization, creatinine level, KDIGO classification from stage 1 to stage 3 (Table S2), suspected drugs. The drugs involved in the included DIAKI cases were classified following the first and second therapeutic level of the International Anatomical, Therapeutic, and Chemical Classification (ATC). For each ATC level 2 classes, we present the five first active substances. The notification rate was calculated for ATC level 2 classes involved in more than 2% for the DIAKI cases and drugs involved in more than 10 DIAKI cases.

Pearson’s chi-squared test for qualitative variables and Student’s *t*-test or Wilcoxon test for quantitative variables were used to compare DIAKI’s characteristics according to whether they have been notified to the RPVC. Statistical significance was defined as a *p*-value lower than 0.05. Statistical analyses were performed using the 3.6.1. version of the R software.

## 3. Results

### 3.1. Hospital Stays Selection

Three thousand seven hundred and fifteen hospital stay with AKI were extracted from the PMSI database. After applying the exclusion criteria, 3641 hospital stays were included in the present study. Among them, 2324 stays met the definition of AKI according to the 2012 KDIGO guidelines, and 2300 stays had an identifiable etiology of AKI. AKI resulting from an adverse drug effect (ADR) was found in 460 stays (20%) (Figure S1). Among the 460 stays, an ADR ICD-10 code (T88.7, Y40, or Y41) was recorded in 80 stays (17.4%). Combining an AKI ICD-10 code with an ICD-10 ADR increased the performance from 12.4% to 28.4%. Among all AKI ICD-10 codes (N17, N99.0, and R39.2), the combination of N99.0 and an ADR ICD-10 code had the best performance (51.1%) for detecting DIAKIs (Table S3).

### 3.2. Pharmacovigilance Cases Selection

During the same period, 91 cases of DIAKI were notified to the RPVC and entered into the FPVDB. After exclusion of cases with no hospitalization (*n* = 7), cases where AKI did not meet the 2012 KDIGO criteria (*n* = 14), cases where AKI was secondary to drug-induced diarrhea (*n* = 3), 67 cases were included in the present study. Among these cases, 59 cases were also present in the PMSI extraction. Eight cases were not found in the PMSI extraction because no AKI code was associated with the corresponding stay (Figure S2).

### 3.3. Estimation of the Prevalence of DIAKIs in Hospital Stays Using Capture-Recapture Method

**Figure 1:**
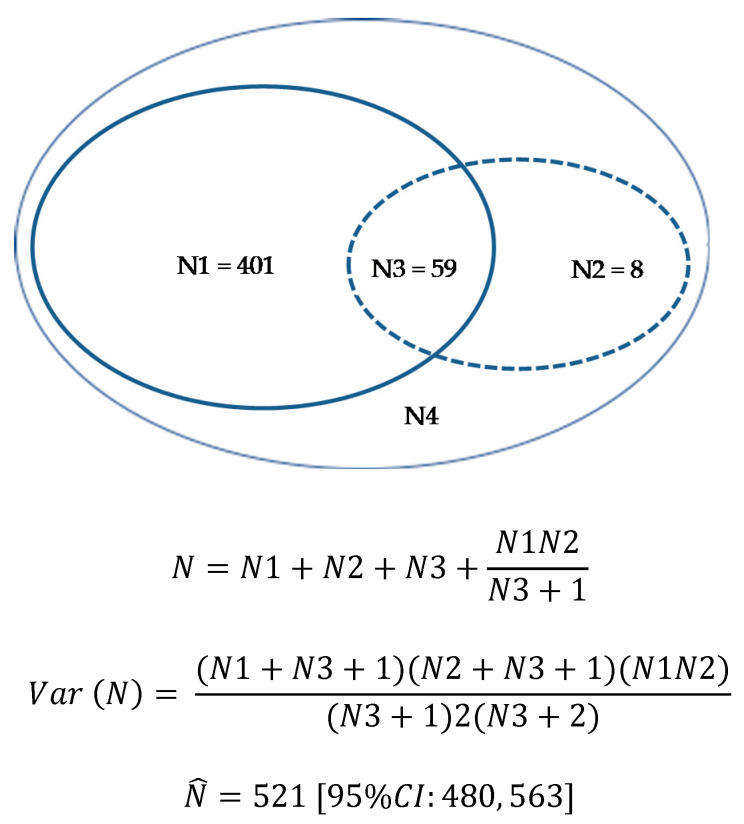
Estimation of the total number of DIAKI using the capture-recapture method. N: total number of DIAKIs; N1: DIAKIs in PMSI database; N2: DIAKIs in FPVDB; N3: DIAKIs common to both databases; N4: DIAKIs are neither coded in the PMSI database nor notified to the pharmacovigilance center. DIAKI: Drug-induced acute kidney injury; FPVDB: French Pharmacovigilance Database; PMSI: French Medical Information System Program.

The PMSI database query detected 460 stays with DIAKI and the FPVDB query 67 cases. Fifty-nine stays were common to both the PMSI database and FPVDB. Using the capture-recapture method, the estimated total number of DIAKIs throughout the study was 521 (95% CI 480, 563) (Figure 1). Considering that 468 DIAKIs were detected out of an estimated total of 521 DIAKIs, we estimated that 53 DIAKIs were not identified (neither by coding nor by notification). The estimated prevalence of DIAKIs within hospital stays was 20.0% (95%CI 18.4, 21.6) of all AKIs (*n* = 2300). The notification rate was 12.9% (95%CI 10.0, 15.8).

### 3.4. Description of the DIAKI Cases

The characteristics of DIAKI cases are reported in Table 1. The mean age of patients with DIAKI was 75.4 (±13.9) years. Most of the patients with DIAKI were male (61.5%). The median duration of the hospitalization was 13 (8–22) days. Regarding medical history, a pre-existing chronic renal disease was present in 22.6% of the included cases and diabetes mellitus in 27.1%. During the stays, acute heart failure was present in 30.3% of included cases and sepsis in 20.9%. Among all DIAKIs cases, two drugs or more were involved in 177 cases (37.8%). According to the KDIGO classification of AKI, 50.2% of the DIAKI cases were at stage 1, 20.3% stage 2, and 29.5% stage 3. Eleven percent of the patients died during the hospital stay and 22.9% required intensive care. RRT was performed for 9.6% of the patients. Among DIAKI cases, the patients who required RRT were significantly younger and sepsis was significantly more often present. Chronic kidney disease, diabetes mellitus, acute heart failure, sex, and the number of drugs suspected did not differ (Table S4).

Compared to non-reported DIAKIs, the patients for whom DIAKI was notified to the RPVC were significantly younger. The reported cases were more severe with significantly longer duration stays, lower kidney function with a higher requirement of intensive care and RRT. Sepsis was more present whereas acute heart failure was less present in reported DIAKI cases by comparison with non-reported DIAKI cases. The mortality rate did not differ between the two groups (Table 1).

### 3.5. Description of the Drug Involved in DIAKIs

**Figure 2:**
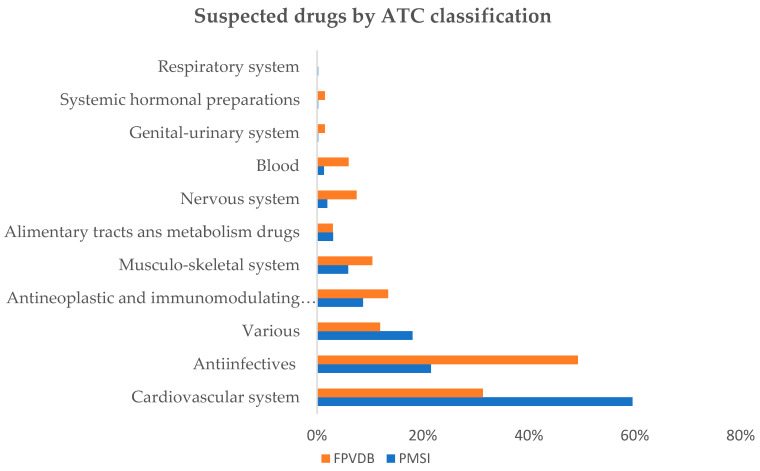
Distribution of ATC classification (1st level) of drugs suspected in DIAKIs according to the data sources. ATC (classification): Anatomical Therapeutic Chemical (classification); DIAKI: Drug-induced Acute Kidney Injury; FPVDB: French Pharmacovigilance Database; PMSI: French Medical Information System Program.

The distribution of the suspected drugs involved in DIAKI is shown in Figure 2 according to the International Anatomical, Therapeutic and Chemical (ATC) Classification level 1. The most frequently suspected drugs were classified under “cardiovascular system”, for the PMSI cases and under “anti-infectives for systemic use” for the FPVDB cases.

Table 2 shows the suspected drugs classified according to ATC level 2. The most represented ATC classes were diuretic drugs, drugs acting on the renin-angiotensin system, antibacterial drugs, contrast media, antineoplastics drugs, anti-inflammatory, and antirheumatic drugs, immunosuppressant drugs, and antiviral drugs.

For these ATC level 2 classes, the highest notification rates were observed for antiviral drugs, antibacterial drugs, followed by antineoplastic drugs and anti-inflammatory and antirheumatic drugs. The lowest notification rates were observed for diuretic drugs, immunosuppressant drugs, and contrast media (Table 2).

## 4. Discussion

Using the capture-recapture method, the present study provides an estimation of the prevalence of DIAKI and the DIAKI notification rate, 20.0% and 12.9%, respectively. The association of ICD-10 codes improves by 4 times the detection of DIAKI in the PMSI database. Using the AKI KDIGO classification, a significant proportion of DIAKI cases are severe and 9.6% required hemodialysis. The most prevalent classes involving in the reported DIAKI cases were diuretic drugs, drugs acting on the renin-angiotensin system, antibacterial drugs, and contrast media.

### 4.1. DIAKIs Prevalence and Notification Rate

The prevalence of DIAKI in the present study is consistent with the literature. Indeed, an estimated rate from 14 to 27% of all AKIs has been reported in various epidemiological studies [5,18,19,20]. However, it is worth noting that the notification rate is still low, close to 10%. In the literature, the notification rate of ADR varies depending on the teaching nature of the hospital [7,21], the setting up of weekly visits to clinical departments, the study population, and the nature of ADRs. Lopez-Gonzalez et al. performed a systematic review of the determinants of ADR under-reporting. Medical specialty, knowledge, and attitudes of health professionals were associated with a better notification rate [22]. In addition, unexpected and severe ADRs are more likely to be notified [23]. In accordance with these observations, the notified DIAKI cases are more severe and involved younger patients.

The use of the PMSI database could provide an interesting tool to improve the detection of DIAKI. In the present study, the exhaustiveness of the PMSI database is 88.3% while that of the FPVDB is 12.9%. Using the PMSI database increased considerably the number of ADRs detected by comparison with spontaneous notifications. The combination of an AKI code and an ADR code improves the ability to detect DIAKIs by up to 51%. This approach can be used in a daily routine to identify and notify DIAKIs.

### 4.2. DIAKI Cases Characteristics

In the studied population, the majority of patients were male and over 60 years old. Half of the DIAKI cases can be considered as severe—KDIGO stages 2 and 3—and hemodialysis was required in 9.6% of cases. The severity of the DIAKI cases is close to that described by Hamzic-Mehmedbasic et al. or Pierson-Marchandise et al. [24,25]. A quarter of included cases had co-morbidities such as diabetes or chronic kidney disease which are recognized risk factors of developing AKI. Lastly, although women were more susceptible to developing AKI, men were more represented in DIAKI studies [11].

### 4.3. Drugs Involved in DIAKI Cases

The repartition of the involved drugs varied depending on the sources of the data. Data from the PMSI database showed that the most prevalent classes were diuretic drugs (41.3%) and drugs acting on the renin-angiotensin system (31.5%). By contrast, the data obtained from the FPVDB involved mainly antibacterial drugs (37.3%) and agents acting on the renin-angiotensin system (22.4%). The data obtained from the FPVDB are consistent with the case-non-case study of Pierson-Marchandise et al. where the most frequently implicated drug classes were antibacterial agents for systemic use (29.5%), diuretics (18.5%), and agents acting on the renin–angiotensin system (16.3%). The use of the PMSI database which has a better exhaustiveness provides a picture closer to reality.

Diuretics are the ATC level 2 classes most involved in all DIAKIs (41.0%) and the most represented in the PMSI database. However, the notification rate of these DIAKIs is low. In acute or chronic pathological situations, diuretic administration is associated with electrolyte and fluid loss [26]. The risk of extracellular dehydration leading to functional renal failure is a well-known ADR controlled by clinicians, which explains the low notification rate.

Agents acting on the renin-angiotensin system including ACEis (Angiotensin-Converting Enzyme inhibitors) and ARBs (angiotensin II-receptor blockers) mainly are the second level 2 ATC classes most involved in all DIAKIs (31.2%) with a notification rate of 10.3%. ARBs and ACEIs have the potential of causing or exacerbating acute renal failure by modulating intra-renal blood flow. ARBs and ACEs decrease intraglomerular pressure through selective inhibition of angiotensin II-mediated vasoconstriction of the efferent arteriole [27,28].

Antibacterial drugs represent 17.9% of all DIAKIs and are the level 2 ATC classes most notified to the RPVC (rate of notification: 29.8%). The three most active substances involved in DIAKIs are amoxicillin (4.5% of all DIAKIs), gentamicin (3.8%) and vancomycin, (3.8%). Sulfamethoxazole/trimethoprim is involved in 3.0% of DIAKI cases but the notification rate is 50.0%. The main mechanism of amoxicillin (AMX) and sulfamethoxazole/trimethoprim (SMX/TMP) that induce renal injury is acute tubule-interstitial nephritis (ATIN), an entity in which acute kidney injury is accompanied by histological findings of interstitial inflammation, edema, and tubulitis [29,30]. Drug-induced ATIN represents two-thirds of all AIN [31]. Antibiotics account for about a third of the cases, penicillins including AMX and sulfonamides including SMX/TMP are particularly involved. Another mechanism of AKI related to AMX and SMX/TMP is crystalluria [32,33]. Regarding the mechanism underlying renal toxicity of vancomycin, recent data suggest that vancomycin causes oxidative effects on the proximal renal tubule resulting in renal tubular ischemia. The drug has also been shown to interfere with the normal reabsorption function of the proximal renal tubule epithelium and alter the mitochondrial function of these cells. Moreover, vancomycin can induce AIN [34]. Gentamicin is an aminoglycoside that induces nephrotoxicity through the endocytosis and accumulation of antibiotics in the epithelial cells of the proximal tubule [35]. Aminoglycoside-induced nephrotoxicity occurs in 10–25% of therapeutic courses [36].

An increase in creatinine levels without kidney injury related to a decrease in creatinine proximal tubular secretion can be observed with drugs such as trimethoprim. Indeed, trimethoprim is an inhibitor of the organic cation transporter 2 (OCT-2), and through this mechanism, decreases tubular creatinine secretion and induces a moderate increase in serum creatinine level (not more than 20%). However, in the present study, the increase in creatinine level observed in the 14 cases of sulfamethoxazole-trimethoprim induced AKI was 20% in one case and was higher than 20% (ranging from 40 to 900%) in 13 cases. Thus, with the exception of one case, the DIAKIs cases involving sulfamethoxazole-trimethoprim are more likely to be due to drug-induced kidney injury than an inhibition of the OCT-2 transporter.

Contrast associated-AKI represent 16.9% of all DIAKIs and the notification rate is 10.1%. It is a well-known cause of AKI and is the third leading cause of hospital-acquired acute renal failure [20]. Many studies have now shown that patients who develop contrast associated-AKI have a greater risk of death or prolonged hospitalization [37]. The pathophysiology of contrast-induced nephropathy (CIN) associates three distinct but interacting mechanisms: medullary ischemia, the formation of reactive oxygen species, and direct tubular cell toxicity [38].

### 4.4. Strengths and Limitations

A rigorous selection of the AKI cases was performed in this study using the definition of the AKI from KDIGO guideline which is an international reference. In addition, all cases were reviewed by a specialist in pharmacovigilance and a nephrologist to obtain high-quality data.

The rules established for coding the activity during inpatient stays vary from country to country. In France, coding of stays is governed by the ATIH (Technical Agency for Information on Hospital Care) which provides a yearly methodological coding guide. For example, the ATIH recommends the use of codes N17 “Acute kidney failure” only in cases of AKI with an organic injury of renal tissue. In cases of functional AKI, code R39.2 “Extrarenal uremia” should be used. On the contrary, in Germany, the German Society of Nephrology recommended coding N17 when AKI met the KDIGO definition [39]. As a consequence, the ability of an association of codes to detect ADR has to be established in each country.

In addition, it is challenging to detect DIAKIs since there is no explicit ICD-10 code to describe a drug-induced AKI while for other clinical situations such a code exists—E06.4 “Drug-induced thyroiditis” or G62.0 “Drug-induced polyneuropathy”, for example. Including such a code in the ICD-10 could help at identifying and studying DIAKI cases.

The extent of the independence of sources cannot be verified statistically when there are only two sources [40]. However, it is common that the coding of the stay using ICD-10 codes and the pharmacovigilance notifications are not performed by the same person.

## 5. Conclusions

In the present study, the prevalence of DIAKI was 20.0% with an estimated notification rate of 12.9%. The combination of N99.0 to an ADR code allows for the identification of more than 50% of DIAKI from the PMSI database. From this study, in addition to spontaneous notifications, a routine request using the association of these ICD-10 codes could be performed to improve DIAKI notification and to improve drug safety surveillance.

## Figures and Tables

**Table 1:** Characteristics of drug-induced kidney injury cases.

	All DIAKIs	DIAKIs Recorded in FPVDB	DIAKIs Not Recorded in FPVDB	
	*n* = 468	*n* = 67	*n* = 401	*p* Value
Age, years [mean ± SD]	75.4 ± 13.9	68.9 ± 13.8	76.5 ± 13.6	<0.001 ^a^
Length of stay, days [median (IQR)]	13 (8–22)	21 (10.5–29.0)	12 (7–20)	<0.001 ^b^
Male [*n* (%)]	288 (61.5)	48 (68.7)	240 (59.9)	0.066 ^c^
Chronic kidney disease [*n* (%)]	106 (22.6)	17 (25.4)	89 (22.2)	0.565 ^c^
Acute heart failure [*n* (%)]	142 (30.3)	12 (17.9)	130 (32.4)	0.017 ^c^
Diabetes mellitus [*n* (%)]	127 (27.1)	17 (25.4)	110 (27.4)	0.726 ^c^
Sepsis [*n* (%)]	98 (20.9)	21 (31.3)	77 (19.2)	0.024 ^c^
Number of drug(s) involved [*n* (%)]				0.037 ^c^
1 drug	291 (62.2)	34 (50.7)	257 (64.1)	
2 drugs or more	177 (37.8)	33 (49.3)	144 (35.9)	
KDIGO stage [*n* (%)]				<0.001 ^c^
Stage 1	235 (50.2)	15 (22.4)	220 (54.9)	
Stage 2	95 (20.3)	11 (16.4)	84 (20.9)	
Stage 3	138 (29.5)	41 (61.2)	97 (24.2)	
Death during stay [*n* (%)]	52 (11.1)	6 (9.0)	46 (11.5)	0.544 ^c^
Intensive care unit hospitalization [*n* (%)]	107 (22.9)	27 (40.3)	80 (20.0)	<0.001 ^c^
Extra-renal replacement therapy [*n* (%)]	45 (9.6)	21 (31.3)	24 (6.0)	<0.001 ^c^

DIAKI: Drug-induced acute kidney injury; FPVDB: French Pharmacovigilance Database; IQR: interquartile range; KDIGO: Kidney Disease Improving Global Outcomes; SD Standard Deviation. ^a^ Student *t*-test; ^b^ Wilcoxon test; ^c^ Pearson Chi-squared test.

**Table 2:** Drug-induced acute kidney injury according to the ATC classification level 2 involved in more than 2% of cases and the 5 most frequent active substances for each class.

ATC Classes	Active Substances	DIAKIs in PSMSI Database *n* = 460		DIAKIs in FPVDB *n* = 67		Total DIAKIs *n* = 468 ^a^		Notification Rate ^b^
		n	%	n	%	n	%	%
**C03**	**Diuretic drugs**	**190**	**41.3**	**11**	**16.4**	**192**	**41.0**	**5.7**
C03CA01	furosemide	167	36.3	6	9.0	168	35.9	3.6
C03AA03	hydrochlorothiazide	17	3.7	3	4.5	18	3.8	16.7
C03DA01	spironolactone	15	3.3	2	3.0	16	3.4	12.5
**C09**	**Agents acting on the renin-angiotensin system**	**145**	**31.5**	**15**	**22.4**	**146**	**31.2**	**10.3**
C09AA05	ramipril	37	8.0	5	7.5	37	7.9	13.5
C09AA04	perindopril	23	5.0	1	1.5	23	4.9	4.3
C09CA04	irbesartan	16	3.5	3	4.5	17	3.6	17.6
C09CA03	valsartan	14	3.0	1	1.5	14	3.0	7.1
C09CA06	candesartan	9	2.0	2	3.0	9	1.9	-
**J01**	**Antibacterial drugs**	**81**	**17.6**	**25**	**37.3**	**84**	**17.9**	**29.8**
J01CA04	amoxicillin	21	4.6	8	11.9	21	4.5	38.1
J01GB03	gentamicin	18	3.9	2	3.0	18	3.8	11.1
J01XA01	vancomycin	18	3.9	2	3.0	18	3.8	11.1
J01EE01	sulfamethoxazole/trimethoprim	14	3.0	7	10.4	14	3.0	50.0
J01GB06	amikacin	8	1.7	1	1.5	8	1.7	-
**V08**	**Contrast media**	**79**	**17.2**	**8**	**11.9**	**79**	**16.9**	**10.1**
V08AB11	iobitridol	52	11.3	6	9.0	52	11.1	11.5
V08AB09	iodixanol	27	5.9	1	1.5	27	5.8	3.7
**L01**	**Antineoplastic drugs**	**21**	**4.6**	**7**	**10.4**	**23**	**4.9**	**30.4**
L01XA02	carboplatin	6	1.3	1	1.5	6	1.3	-
L01CB01	etoposide	5	1.1	1	1.5	6	1.3	-
L01AA01	cyclophosphamide	4	0.9	1	1.5	5	0.9	-
L01XA01	cisplatin	4	0.9	1	1.5	4	0.9	-
L01BA01	methotrexate	4	0.9	0	0.0	4	0.9	-
**M01**	**Anti-inflammatory and antirheumatic drugs**	**19**	**4.1**	**4**	**6.0**	**19**	**4.1**	**21.1**
M01AE03	ketoprofen	7	1.5	1	1.5	7	1.5	-
M01AE13	ibuprofen	3	0.7	1	1.5	3	0.6	-
**L04**	**Immunosuppressant drugs**	**16**	**3.5**	**2**	**3.0**	**17**	**3.6**	**11.7**
L04AD02	tacrolimus	9	2.0	0	0.0	9	1.9	-
L04AD01	cyclosporine	5	1.0	0	0	5	1.1	-
L04AX04	lenalidomide	1	0.2	0	0.0	1	0.2	-
**J05**	**Antiviral drugs**	**14**	**3.0**	**7**	**10.4**	**15**	**3.2**	**46.7**
J05AB01	acyclovir	8	1.7	4	6.0	8	1.7	-
J05AB11	valaciclovir	3	0.7	1	1.5	3	0.6	-

ATC classification: Anatomical Therapeutic Chemical classification; DIAKI: Drug-induced acute kidney injury; FPVDB: French Pharmacovigilance Database; PMSI: French Medical Information System Program; ^a^ 59 DIAKI cases are common in PMSI database and FPVDB; ^b^ The notification rate was calculated for active substances implicated in at least 10 DIAKIs.

## Data Availability

The data presented in this study are available on request from the corresponding author. The request should be accompanied by a research protocol. The data are not publicy available due to European ethical and legal restrictions.

## Supplementary Materials

The following are available online at https://www.mdpi.com/2077-0383/10/2/168/s1; Table S1: ICD-10 codes used for the PMSI database query, Table S2: KDIGO—AKI Classification; Table S3: Performance of PMSI database queries to detect DIAKIs; Table S4: Characteristics of patients who required renal replacement therapy; Figure S1: Flowchart of the extraction of stays with DIAKI from the PMSI database; Figure S2: Flowchart of the extraction of stays with DIAKI from the FPVDB.
