# Supplementary material for: Drug-Induced Acute Kidney Injury: A Study from the French Medical Administrative and the French National Pharmacovigilance Databases Using Capture-Recapture Method
Source: J Clin Med. 2021 Jan 6;10(2):168. doi: 10.3390/jcm10020168 (PMC7824808; doi:10.3390/jcm10020168)
Supplement: Supplementary file 1 [file jcm-10-00168-s001.pdf]

Electronic Supplementary Materials
Drug Safety

Drug-Induced Acute Kidney Injury: A Study from the French Medical Administrative and the French Pharmacovigilance Databases using Capture-Recapture Method

Authors: Rolland AL[1], Garnier AS[2,4], Meunier K[1], Drablier G[3], Briet M[3,4]

[1]Département d'Information Médicale, Centre Hospitalo-Universitaire d'Angers, Angers, France, [2]Service de Néphrologie-Dialyse-Transplantation, Centre Hospitalo-Universitaire d'Angers, Angers, France, [3] Laboratoire de Pharmacologie-Toxicologie et Centre Régional de Pharmacovigilance, Centre Hospitalo-Universitaire d'Angers, Angers, France, 4Laboratoire MitoVasc, INSERM U1083, CNRS UMR 6015, Université d'Angers, Angers, France

Corresponding author: Prof. Marie Briet
Address : Service de Pharmacologie-Toxicologie et Pharmacovigilance, Centre Hospitalo-Universitaire d'Angers, 4 rue Larrey, 49100 ANGERS
E-mail :marie.briet@chu-angers.fr

Supplementary figures

Figure S1 Flowchart of the extraction of stays with DIAKI from the PMSI database

Figure S2 Flowchart of the extraction of stays with DIAKI from the FPVDB

Supplementary tables

Table S1 ICD-10 codes used for the PMSI database query

Table S2 KDIGO - AKI Classification

Table S3 Performance of PMSI database queries to detect DIAKIs

Table S4 Characteristics of patients who required renal replacement therapy

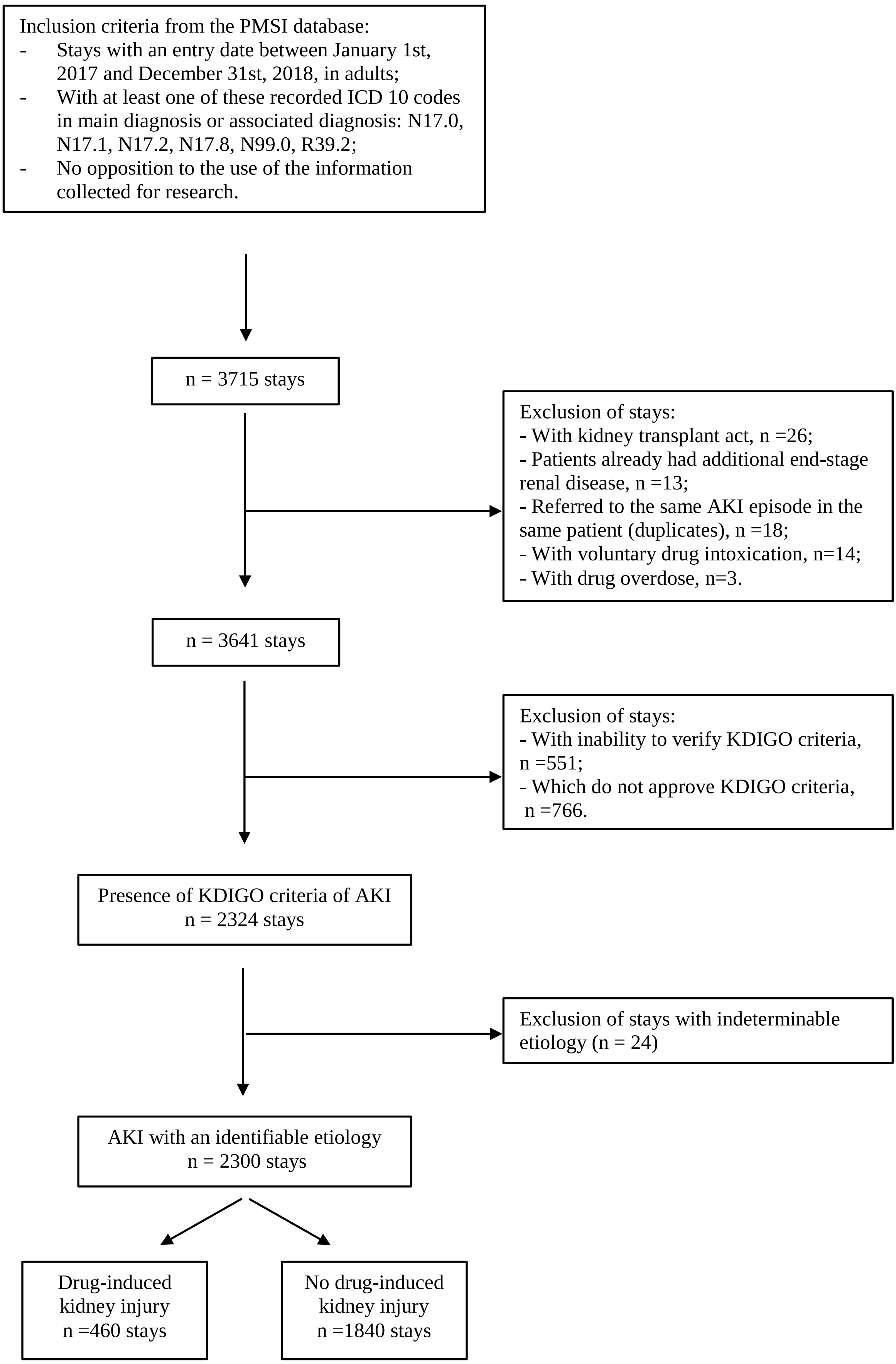
Inclusion criteria from the PMSI database:
- Stays with an entry date between January 1st, 2017 and December 31st, 2018, in adults;
- With at least one of these recorded ICD 10 codes in main diagnosis or associated diagnosis: N17.0, N17.1, N17.2, N17.8, N99.0, R39.2;
- No opposition to the use of the information collected for research.
n = 3715 stays
Exclusion of stays:
- With kidney transplant act, n =26;
- Patients already had additional end-stage renal disease, n =13;
- Referred to the same AKI episode in the same patient (duplicates), n =18;
- With voluntary drug intoxication, n=14;
- With drug overdose, n=3.
n = 3641 stays
Exclusion of stays:
- With inability to verify KDIGO criteria, n =551;
- Which do not approve KDIGO criteria, n =766.
Presence of KDIGO criteria of AKI
n = 2324 stays
Exclusion of stays with indeterminable etiology (n = 24)
AKI with an identifiable etiology
n = 2300 stays
Drug-induced kidney injury
n =460 stays
No drug-induced kidney injury
n =1840 stays

Figure S1 Flowchart of the extraction of stays with DIAKI from the PMSI database
AKI: Acute Kidney Injury; drug-induced acute kidney injury; ICD 10: International Statistical Classification of Diseases 10th Revision; KDIGO: Kidney Disease Improving Global Outcomes; PMSI: French Medical Information System Program

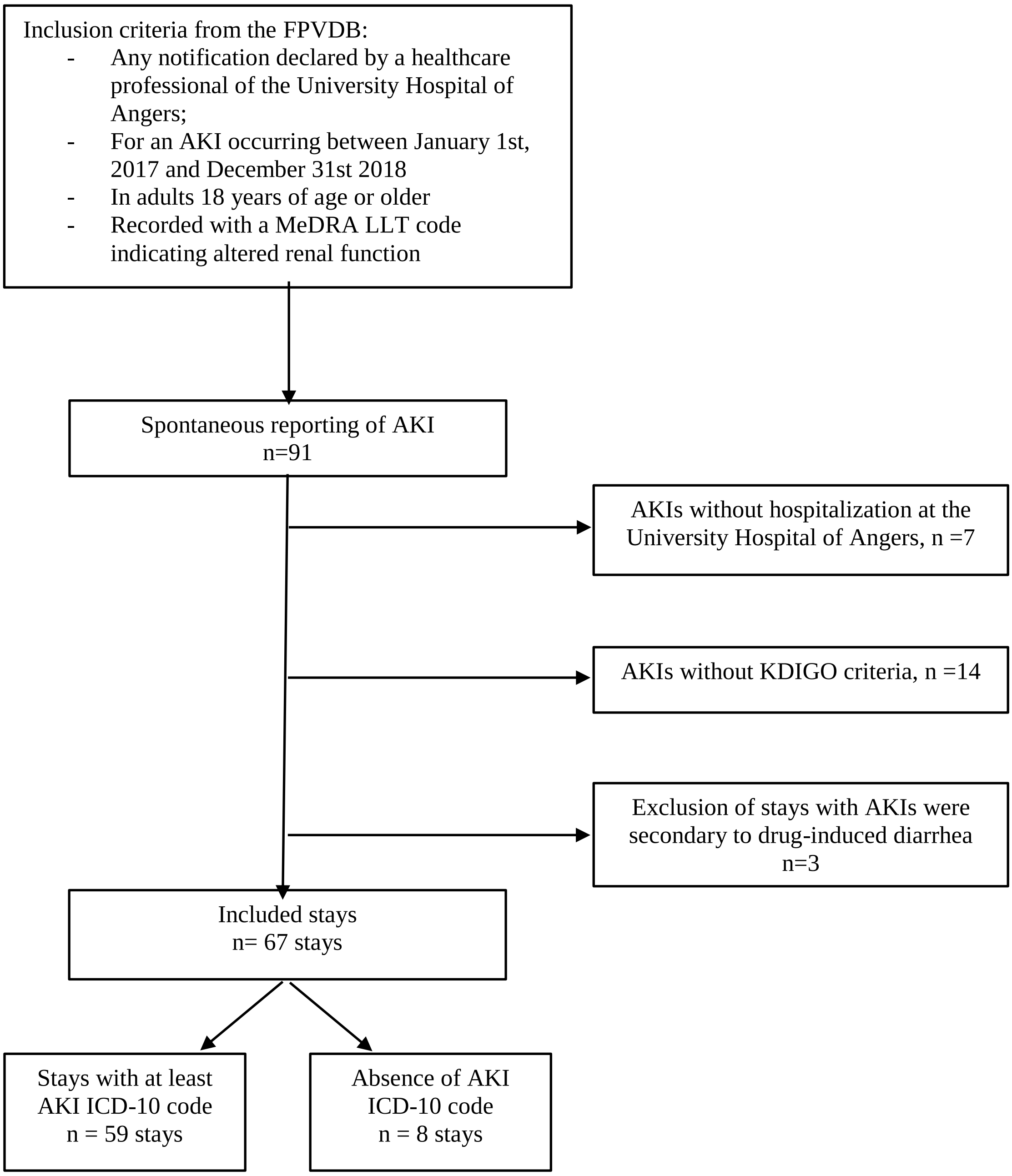

Figure S2 Flowchart of the extraction of stays with DIAKI from the FPVDB request
AKI: Acute Kidney Injury; drug-induced acute kidney injury; ICD 10: International Statistical Classification of Diseases 10th Revision; KDIGO: Kidney Disease Improving Global Outcomes; FPVDB: French Pharmacovigilance Database.

Table S1 ICD-10 codes used for the PMSI database query[1]

ICD-10 Codes	Diagnosis
N17.0	Acute renal failure with tubular necrosis
N17.1	Acute renal failure with acute cortical necrosis
N17.2	Acute renal failure with medullary necrosis
N17.8	Other acute renal failure
N17.9	Acute renal failure, unspecified
N99.0	Postprocedural renal failure
R39.2	Extrarenal uraemia

ICD-10: International Classification of Diseases 10th Revision; PMSI: French Medical Information System Program

Table S2 KDIGO - AKI Classification[2]

Stage	Creatinine Criteria	Urine Output Criteria
1	Creatinine 1.5-1.9 times baseline,<br>OR<br>Creatinine increase >0,3mg/dL (≥26.5μmol/l) increase	<0.5ml/kg/h for 6-12 hours
2	2.0-2.9 times baseline	<0.5ml/kg/h for ≥12 hours
3	3.0 times baseline<br>OR<br>Increase in serum creatinine to ≥4.0mg/dl (≥353.6μmol/l)<br>OR<br>Initiation of renal replacement therapy<br>OR, in patients <18 years, decrease in eGFR to <35ml/min per 1.73 m^2	<0.3 m/kg/h for ≥24 hours<br>OR<br>Anuria for ≥12 hours

AKI: Acute Kidney Injury; KDIGO: Kidney Disease Improving Global Outcomes; SCr: Serum creatinine

Table S3 Performance of PMSI database queries to detect DIAKIs

	DIAKI N=460	Total sample[a] N=3715	Performance[b]
**ICD-10 codes**	**N**	**N**	**%**
AKI code[c]	460	3715	12,4
AKI code + ADR code[d]	80	282	28,4
N17	158	1247	12,7
N17. + ADR code	32	98	32,7
N99.0	128	825	15,5
N99.0 + ADR code	23	45	51,1
R39.2	219	1868	11,7
R39.2+ ADR code	37	171	21,6

ADR : Adverse Drug Reaction; AKI: Acute Kidney Injury; DIAKI: Drug-induced Acute Kidney Injury; ICD-10: International Classification of Diseases 10th Revision; Code N17: Acute renal failure; Code N99: Postprocedural renal failure; Code R39.2: Extrarenal uraemia; PMSI: French Medical Information System Program.

[a]Several stay summaries included more than one AKI ICD-10 code.
[b]Performance is defined by the percentage of DIAKIs detected by the ICD-10 codes query.
[c]AKI ICD-10 codes are N17., N99.0 and R39.2.
[d]ADR ICD-10 codes are T88.7, and codes starting with Y40. and Y41.

Table S4 Characteristics of patients who required renal replacement therapy

	RRT n=45		No RRT n=423		
	**N**	**%**	**N**	**%**	**P Value[a]**
Age >75 years	16	35.6	243	57.4	0.005
Male	32	71.1	256	60.5	0.165
Chronic kidney disease	9	20.0	97	22.0	0.656
Heart failure	11	24.4	131	31.0	0.365
Diabetes mellitus	12	26.7	115	27.2	0.941
Sepsis	19	42.2	79	18.7	<0.001
Two drugs or more suspected in DIAKIs	19	42.2	158	37.4	0.632

DIAKI: Drug-induced Acute Kidney Injury; RRT: renal replacement therapy
[a]Pearson chi-square tests

References

1. ICD-10 Version:2019. https://icd.who.int/browse10/2019/en#/. Accessed 5 Nov 2020

2. KDIGO Clinical Practice Guideline for Acute Kidney Injury (2012). Kidney International Supplements 2:1
